# Roles of Krüppel‐like factor 5 in kidney disease

**DOI:** 10.1111/jcmm.16332

**Published:** 2021-02-01

**Authors:** Jia Li, Liang Liu, Wen‐Qian Zhou, Lu Cai, Zhong‐Gao Xu, Madhavi J. Rane

**Affiliations:** ^1^ Department of Nephrology The First Hospital of Jilin University Changchun China; ^2^ Department of Pediatrics Pediatric Research Institute University of Louisville Louisville KY USA; ^3^ Department of Radiology China‐Japan Union Hospital of Jilin University Changchun China; ^4^ The Center of Cardiovascular Diseases The First Hospital of Jilin University Changchun China; ^5^ Department of Pharmacology and Toxicology University of Louisville Louisville KY USA; ^6^ Department of Medicine Division of Nephrology Department of Biochemistry and Molecular Genetics University of Louisville Louisville KY USA

**Keywords:** diabetes, fibrosis, inflammation, kidney disease, Krüppel‐like factor 5, Krüppel‐like factors

## Abstract

Transcription factor Krüppel‐like factor 5 (KLF5) is a member of the Krüppel‐like factors’ (KLFs) family. KLF5 regulates a number of cellular functions, such as apoptosis, proliferation and differentiation. Therefore, KLF5 can play a role in many diseases, including, cancer, cardiovascular disease and gastrointestinal disorders. An important role for KLF5 in the kidney was recently reported, such that KLF5 regulated podocyte apoptosis, renal cell proliferation, tubulointerstitial inflammation and renal fibrosis. In this review, we have summarized the available information in the literature with a brief description on how transcriptional, post‐transcriptional and post‐translational modifications of KLF5 modulate its function in a variety of organs including the kidney with a focus of its importance on the pathogenesis of various kidney diseases. Furthermore, we also have outlined the current and possible mechanisms of KLF5 activation in kidney diseases. These studies suggest a need for more systemic investigations, particularly for generation of animal models with renal cell‐specific deletion or overexpression of *KLF5* gene to examine direct contributions of KLF5 to various kidney diseases. This will promote further experimentation in the development of therapies to prevent or treat various kidney diseases.

## 1 INTRODUCTION

Kidney disease is a major global health issue and is associated with a tremendous economic burden. However, the pathogenesis of kidney disease is currently not fully understood, and there are currently no effective treatments. Therefore, it is important to identify new effective therapeutic targets to treat various kidney diseases.^1^, ^2^

Transcription factor Krüppel‐like factor 5 (KLF5) is a member of the Krüppel‐like factors (KLFs) family.^3^ KLF5 was originally known as ‘basic transcription element‐binding protein 2 (BTEB2)’ or ‘colon Krüppel‐like factor (CKLF)’ or ‘intestinal enriched Krüppel‐like factor (IKLF)’.^4^, ^5^ In the 1990s, the Human Gene Nomenclature Committee, reclassified the KLF nomenclature and uniformly named all the KLFs in the order of their discovery, so it has the name KLF5.^6^ Human *KLF5* gene is located on chromosome 13q22.1. It encodes for a 457 amino acid protein in humans. Its predicted molecular weight is about 51 kDa. Mouse Klf5 protein contains 446 amino acids, with predicted molecular mass of 50 kDa and its *Klf5* gene is located on chromosome 14 (Table 1).^5^, ^7^ KLF5 has three highly conserved C2H2 type zinc‐finger domains in its C‐terminal known to bind GC‐rich regions in DNA, leading to modulation of their gene targets.^8^

**TABLE 1:** Comparing basic features of KLF5 between human and mouse (based on Ensembl)

Species	Human	Mouse
Protein name	Krüppel‐like factor 5 (KLF5)	Krüppel‐like factor 5 (Klf5)
Gene Synonyms	BTEB2, CKLF, IKLF	4930520J07Rik, Bteb2, CKLF, IKLF
Location	13q22.1 (Chromosome 13:73054976‐73077541)	14 (Chromosome 14:99298691‐99315036)
Gene type	Protein coding	Protein coding
Size of protein	457 amino acids	446 amino acids
Molecular Mass of protein (Da)	50 792 Da	49 754 Da
Expression	Skin, oesophagus, colon, small intestine and kidney. (HPA RNA‐seq normal tissues)	Colon, duodenum, small intestine, stomach and kidney. (Mouse ENCODE transcriptome data)
Ensembl	ENSG00000102554	ENSMUSG00000005148
UniProt	Q13887	Q9Z0Z7

Abbreviations: BTEB2, basic transcription element‐binding protein 2; CKLF, colon Krüppel‐like factor; Da, Dalton; ENCODE, encyclopaedia of DNA elements; HPA, Human Protein Atlas; IKLF, intestinal enriched Krüppel‐like factor.

KLF5 regulates a number of cellular functions and can play a part in a variety of diseases, such as cancer, cardiovascular disease and gastrointestinal disorders.^8^ Recently, KLF5 was identified in the kidney and was shown to regulate podocyte apoptosis, renal cell proliferation, tubulointerstitial inflammation and renal fibrosis.^9^, ^10^, ^11^, ^12^, ^13^ In this review, we have summarized the current evidence confirming an important role for KLF5 in various kidney diseases and we have discussed the possible mechanisms regulating KLF5 expression transcriptionally or translationally. Moreover, we have outlined the possible pathogenic pathways that are involved in the kidney diseases, including, acute kidney injury (AKI) and chronic renal fibrosis related to nephropathy and nephritis. The mechanistic studies that precisely define KLF5 regulation of kidney diseases are not completed known. Therefore, in the current review, we have included mechanisms that regulate KLF5 stability and/or activity in other organs and cell types in addition to the kidney. These studies will drive further research in delineating the mechanistic role of KLF5 in kidney diseases. These studies collectively suggest that KLF5 may serve as a new therapeutic target in the treatment of kidney disease.

## 2 POST‐TRANSLATIONAL, TRANSCRIPTIONAL, POST‐TRANSCRIPTIONAL REGULATION OF KLF5

Post‐translational, transcriptional and post‐transcriptional regulation are main regulatory mechanisms of KLF5 expression. Acetylation, phosphorylation, ubiquitination and sumoylation are some post‐translational modifications that regulate stability and activity of a variety of proteins, including KLF5.^14^ Transcriptional regulation is the modifications that occur when DNA is transcribed into RNA, including control at the level of transcription apparatus, transcription factors and chromatin.^15^ These aforementioned modifications of KLF5 often occur by recruitment of modifier proteins (co‐activators or co‐repressors) by KLF5 and can regulate KLF5‐mediated target gene expression. Post‐transcriptional regulation are the modifications that occur after DNA is transcribed into RNA and before RNA is translated into protein, including modifications of RNA splicing, nuclear export, RNA stability and miRNA‐mediated regulation of protein expression.^16^, ^17^ Although, the role for KLF5 in mediating kidney disease is established, the studies involving KLF5 transcriptional, post‐transcriptional and post‐translational modifications in the kidney are limited. Therefore, in order to better understanding the role of KLF5 in the pathogenesis of various kidney diseases and to drive future studies in the kidney, we have summarized how KLF5 is regulated post‐translationally, transcriptionally and post‐transcriptionally in the various cells and tissues, including in the kidney.

### 2.1 Acetylation

Acetylation and deacetylation occur *via* addition or removal of an acetyl group (CH_3_CO‐) on a protein. Changes in protein acetylation can alter its function.^18^, ^19^ KLF5 is a pro‐proliferative transcription factor, known to promote cellular proliferation by inhibiting p15 or cyclin‐dependent kinase inhibitor 2B (CDKN2B) expression. However, in the presence of transforming growth factor beta (TGF‐β), p300 was recruited to the KLF5‐SMAD complex, which acetylated KLF5. Acetylation of KLF5 altered binding of other factors to p15 promoter resulting in induction of p15 expression and inhibition of cellular proliferation in various cell types including, human embryonic kidney cell line (HEK293) cells.^20^, ^21^, ^22^ Acetylation of KLF5 by p300 and deacetylation by histone deacetylase 1 (HDAC1) and histone chaperone TAF1/SET is also reported in vascular smooth muscle cells (VSMCs) and in human HeLa cells.^23^, ^24^ Acetylation of KLF5 at K369 by p300 enhanced its transactivation activity,^23^ while HDAC1 mediated KLF5 deacetylation inhibited its transactivation activity.^24^

### 2.2 Phosphorylation/ubiquitination

Resveratrol induced KLF5 phosphorylation in renal HEK293 cells and prevented association of KLF5 with c‐Myc.^25^ Moreover, KLF5 phosphorylation could modulate its activation status as KLF5‐Ser153 phosphorylation by protein kinase C at the CREB binding protein (CBP) interaction site promoted its transactivation function in human epithelial cells (HEC‐1B),^26^ while angiotensin II (Ang II)‐induced extracellular signal‐regulated kinase (ERK)‐mediated KLF5 phosphorylation promoted interaction of KLF5 and c‐Jun, resulting in the suppression of p21 expression in VSMCs.^27^ In addition, KLF5 phosphorylation could also modulate its stability, as GSK‐3β induced phosphorylation of KLF5, was shown to promote FBW7‐mediated KLF5 degradation.^28^ A pulse‐chase experiment revealed that KLF5 was a rapid turnover protein with a half‐life of approximately 2 hours. KLF5 was ubiquitinated and degraded at the proteasome in epithelial cells.^29^ WWP1 E3 ubiquitin ligase bound via its WW domain to the PY motif in the transactivation domain on KLF5, resulting in the ubiquitination and degradation of KLF5.^30^ In contrast, ubiquitin independent proteasomal degradation of KLF5 was also documented.^31^

### 2.3 Sumoylation

Sumoylation is a protein modification, which can alter protein stability, protein function/activity, protein cellular localization and appropriate protein targeting.^14^ Sumoylation occurs when a small ubiquitin‐related modifier (SUMO) is incorporated in a protein. This modification can alter the function of metabolic enzymes and/or the metabolic pathways by modulating functions of key transcription factors.^32^ KLF5 is a critical regulator of energy metabolism and its sumoylation is documented.^33^, ^34^ Sumoylation of KLF5 acts as a molecular switch that controls its association with either transcriptional activation or transcriptional repression complexes.^34^ KLF5 regulated lipid metabolism by activating peroxisome proliferator‐activated receptor‐δ (PPAR‐δ) pathway. KLF5 was basally sumoylated, which promoted its association with repressor complexes, and inhibited expression of lipid oxidation genes. Upon agonist binding to PPAR‐δ, KLF5 became desumoylated resulting in its association with an activation complex, which induced expression of lipid metabolism genes. Thus, KLF5 sumoylation status controlled its switch between transcriptional repression to activation.^35^ In addition, KLF5 sumoylation also controlled its nuclear localization.^33^ Du et al further identified K151 and K202 as sumoylation sites on KLF5. KLF5 sumoylation facilitated its nuclear localization and function by inactivating its nuclear export signal.^33^

### 2.4 Methylation

Adding methyl groups (‐CH_3_) to DNA is the progress of DNA methylation, which usually leads to transcriptional silencing.^36^ KLF5 methylation was reported in several cells/tissues and diseases. To date, a single study which demonstrated that in clear cell renal cell carcinoma (ccRCC), KLF5 protein expression was lower in tumour tissues as compared to adjacent normal renal tissues and was also lower in different ccRCC (A498, RCC4 and 786‐O) cell lines compared to immortal renal HEK‐293T cells.^37^ The reduced expression of KLF5 was found to be associated with increased methylation of CpG loci in the promoter of KLF5 gene in the ccRCC or cell lines compared to normal renal tissue or normal cell lines. Furthermore, treatment of ccRCC cells with 5‐Aza‐CdR, a DNA methyltransferase (DNMT) inhibitor, up‐regulated the expression of KLF5 and repressed ccRCC cell growth, suggesting that hypermethylation might contribute to the down‐regulation of KLF5 in ccRCC.^37^ This epigenetic modification of KLF5 was found in renal tumour tissues and cell lines. This study indicated that KLF5 was expressed in renal tissue and cells and its expression was controlled by its DNA methylation levels of gene promoter. Similarly, in dermal fibroblasts of systemic sclerosis, CpG methylation of KLF5 promoter contributed to down‐regulation of KLF5 protein.^38^ Hypermethylation of KLF5 intron 1 was also associated with decreased KLF5 expression in acute myeloid leukaemia and was related to poor overall survivial.^39^

### 2.5 Effects of miRNAs, activators, repressors on KLF5 gene expression

Identifying pathogenic roles of microRNAs (miRNAs) could have an important clinical impact for treating and preventing kidney diseases. Eventually, this could lead to novel and specific therapies and diagnostic tools for kidney diseases. Several studies have suggested that microRNAs, namely, miR‐145, miR‐152, miR‐10b‐3p, miR‐448‐3p, miR‐375 and miR‐9, can suppress the expression of KLF5 by directly interacting with its 3’‐untranslated regions.^40^, ^41^, ^42^, ^43^, ^44^, ^45^, ^46^ MiR‐145‐5p can also target and inhibit KLF5 expression.^47^ MiR‐145 was detected in urinary exosomes of type 1 diabetic patients and in experimental models of diabetes, and its expression was increased in the glomeruli of diabetic animals.^48^ Moreover, MiR‐145 inhibited KLF5‐NFκB‐inflammation pathway in lipopolysaccharide (LPS) treated macrophages.^40^, ^41^ Additionally, smooth muscle enriched long noncoding RNA competitively bound to miR‐10b‐3p and exerted an inhibitory effect on miR‐10b‐3p‐KLF5 pathway in atherosclerosis model.^42^ MiR‐152 targeted KLF5 and suppressed the inflammatory responses in atherosclerosis model.^43^ Furthermore, miR‐488‐3p also targeted KLF5 and promoted an anti‐inflammatory pathway in macrophages in a rat model of intracranial aneurysm.^44^ In the progression of oral squamous cell carcinoma, KLF5 regulated genes involved in proliferation and apoptosis. MiR‐375 repressed KLF5 activation and resulted in abrogation of cellular proliferation and induction of cell apoptosis.^45^ KLF5 expression and activation was inhibited in VSMCs treated with high glucose and miR‐9 mimic, while, in the presence of miR‐9 inhibitor, the expression of KLF5 was increased compared to cells treated with high glucose alone. Moreover, expression and activation of KLF5 was associated with proliferation and migration of VSMCs.^46^

KLF5 could also be regulated by many other activators and repressors which are related to kidney disease. As we know, Ang II and TNF‐α are important mediators in kidney disease. Ang II can lead to hemodynamic effects and activate inflammation/fibrosis pathways in kidney disease.^49^ TNF‐α is a pro‐inflammatory cytokine in kidney disease.^50^ It was reported that human VSMCs treated with either Ang II or TNF‐α induced KLF5 expression along with a unique inhibitor of apoptosis protein‐survivin. Overexpression of survivin also led to up‐regulation of KLF5 in human VSMCs.^51^ Moreover, CCAAT/enhancer‐binding proteins (C/EBP) β and δ were shown to induce KLF5 expression during adipocyte differentiation of mouse embryonic fibroblasts^52^ and C/EBP‐β was also a regulator of kidney disease. Overexpression of C/EBPβ in the kidney of ischaemia reperfusion‐injured mice aggravated the kidney injury, it increased the level of blood urea nitrogen (BUN) and creatinine.^53^ In addition, Egr‐1, a reported factor that promoted kidney injury, was demonstrated to activate KLF5 in VSMCs.^54^, ^55^ Furthermore, IL‐1β, HIF‐1α and C3a, known regulators of kidney disease, could induce KLF5 expression.^56^, ^57^ In contrast, overexpression of C/EBP‐α inhibited KLF5 and decreased invasiveness of the human colon cancer cells (SW480 cells).^58^ Similarly, C/EBP‐α was shown to have a protective effect in the podocytes of mice subjected to Adriamycin‐induced kidney injury as knockout of C/EBP‐α aggravated Adriamycin‐induced kidney injury.^59^ All‐trans retinoid acid was shown to repress KLF5 expression in intestinal epithelial cells (IEC6)^60^ and was shown to have an anti‐inflammatory effect on diabetic kidney disease.^61^ Moreover, sex hormones were known to influence kidney disease.^62^ It was reported that androgen‐induced KLF5 expression in human breast cancer cell lines and a prodrug of 17β‐oestradiol inhibited KLF5‐NFκB inflammatory pathway in the Alzheimer's Disease mouse model.^63^, ^64^ In addition, oncogenic regulator protein SET inhibited KLF5 activation by binding to its DNA‐binding domain (DBD). SET binding prevented KLF5 DBD acetylation by its coactivator/acetylase p300. In the absence of SET, p300 acetylated DBD of KLF5 and activated its transcription.^23^ The Human Protein ATLAS demonstrated that the SET protein was expressed in many tissues including the kidney. Additionally, overexpression of SET in human embryonic kidney 293T cells promoted the cell proliferation.^65^

## 3 EVIDENCE FOR THE ROLES OF KLF5 IN MODELS OF KIDNEY DISEASE

The earliest study on the role of KLF5 in the kidney was found in 2004.^66^ To date, several lines of evidence have identified the role of KLF5 in various animal and cell models of kidney diseases, for which the key information is summarized in Table 2. From these studies, it is appreciated that KLF5 is mainly expressed in the nucleus of renal collecting duct epithelial cells of normal mice.^11^, ^12^, ^13^ However, KLF5 expression is also induced or increased in other renal cells, such as proximal tubule cells, distal tubule cells and mesangial cells of mice under different pathogenic conditions (Table 2).^10^, ^11^, ^12^, ^13^, ^67^ Renal fibrosis and dysfunction are the two common hallmarks of progressive renal diseases.

**TABLE 2:** Current evidence for the roles of KLF5 in models of kidney disease

Disease models	Regulation of KLF5 expression	The role of KLF5 in kidney	Refs
PAN‐induced podocyte injury model.	Overexpress KLF5 in podocytes.	KLF5 overexpression inhibits PAN‐induced podocyte cell cycle arrest and cellular apoptosis by regulating expression of apoptosis‐related proteins and by inhibiting ERK and p38 MAPK activation.	9
Db/db mouse model of DN. LPA‐treated mouse mesangial cell model.	↑in renal cortex of db/db mice (9 wk of age). ↑in LPA (6 h) treated mesangial cells ↓in LPA (12h) treated mesangial cells ↓by KLF5 siRNA	LPA stimulated Rac1/MAPK(p38)/Egr1/KLF5 pathway to increase cyclin D1 expression while decreasing p27^Kip1^ expression, leading to proliferation of mesangial cells. Knocking down KLF5 by KLF5 siRNA increased p27^Kip1^ expression in presence of LPA	10
C57BL/6 mouse model of 5/6Nx‐induced CKD. High‐dose MK‐treated human proximal tubular epithelial cell (HK‐2) model of CKD.	↑in fibrotic mouse kidney and high‐dose MK‐stimulated HK‐2 cells.	KLF5 promoted kidney fibrosis through activation of HIF‐1α‐KLF5‐TGF‐β1 pathway.	11
UUO‐induced renal tubulointerstitial damage in Klf5^+/−^ and control (C57BL6/6J) mice.	↑ in the renal collecting duct cells after UUO.	KLF5^+/−^ mice were protected against UUO‐induced kidney injury by blocking renal cell inflammation and apoptosis. However, KLF5 haplodeficiency induced renal fibrosis suggesting an anti‐fibrotic role for KLF5.	12
C57BL/6 mouse model of UUO and 5/6 Nx‐induced fibrotic kidneys. Mouse renal proximal tubule cells cultured on stiff matrix‐model of CKD.	↑ after UUO and 5/6 Nx in mice as well as in mouse renal proximal tubule cells cultured on stiff matrix.	KLF5 promoted renal tubular cell proliferation through activation of ERK/YAP1/KLF5/cyclin D1 pathway. KLF5 modulates renal fibrosis pathways.	13
BALB/c nude mouse model of ccRCC. Human ccRCC cell model. Tumour and adjacent control tissues of ccRCC patients.	↓ in ccRCC tissues of patients.	Methylated KLF5 suppressed the proliferation and migration of ccRCC cells. DNMT‐1 induced KLF5 methylation which inhibited its expression; however, this effect was reversed by 5‐Aza‐CdR, which is inhibitor of DNMT‐1.	37
C3H/HenAf‐nu^+^ mouse model of radiation‐induced kidney injury.	↑in kidneys 10, 20 wk after irradiation	KLF5 mRNA level was induced in kidneys after 10‐, 20‐ week‐irradiation along with renal structural capillary injury.	66
ccRCC cell model	↑ in the cholesterol treated ccRCC cells	Increased KLF5 expression activated miR‐27a which suppressed FBXW7 expression. This signalling pathway enhanced migration and invasion of ccRCC cells.	67
C57BL/6‐C3‐deficient and WT mouse model of UUO‐induced kidney injury. Complement C3 treated mouse renal tubular epithelial cell (TCMK‐1) model of kidney injury.	↑ after UUO in WT mice ↓ in C3‐deficient UUO mice compared with WT mice subjected to UUO ↑ in C3a‐treated TCMK‐1 cells	Complement C3 up‐regulated KLF5 mRNA level with concurrent increase in EMT, which in turn activated renin and promoted kidney injury. In UUO‐treated C3‐deficient mice, there were decreased KLF5 mRNA expression with concurrent decrease in EMT	69
WT and C3 knockout SHR model of essential hypertension.	↑in renal mesangial cells from SHR compared to control rats ↓in renal mesangial cells from C3 knockout SHR as compared to WT SHR.	In WT SHR, KLF5 mRNA level in renal mesangial cells increased with kidney injury. In C3 knockout SHR, KLF5 mRNA level in renal mesangial cells decreased with alleviated kidney injury.	71
Sprague‐Dawley rat model of melamine‐cyanurate crystal‐induced nephritis.	↑in the melamine‐cyanurate crystal‐induced nephritis	KLF5 mRNA level increased along with the up‐regulation of pro‐inflammatory cytokines.	72
Human biopsy samples from kidney allografts before transplantation and 0, 3, 12 mo after transplantation.	↑in the biopsy samples from kidney allografts after transplantation	KLF5 gene was one of the genes markedly up‐regulated after transplantation as early response gene to ischaemia reperfusion injury	74
Peripheral blood mononuclear cells from children with nephrotic syndrome.	↓in the peripheral blood mononuclear cells of children with nephrotic syndrome	KLF5 mRNA level decreased in the peripheral blood mononuclear cells from children with nephrotic syndrome as compared to control individuals.	75

Abbreviations: ↑ increase; ↓ decrease; 5/6Nx, 5/6 nephrectomy; ccRCC, human clear cell renal cell carcinoma; CKD, chronic kidney disease; DN, diabetic nephropathy; DNMT‐1, DNA methyltransferase 1; EMT, epithelial‐mesenchymal transition.ERK, extracellular signal‐regulated kinase; LPA, lysophosphatidic acid; MAPK, mitogen‐activated protein kinase; MK, MK8617, a kind of hypoxia‐inducible factor (HIF)‐prolyl hydroxylase inhibitor (PHI); PAN, puromycin aminonucleoside; SHR, spontaneously hypertensive rats; TGF‐β, transforming growth factor beta; UUO, unilateral ureteral obstruction; WT, wide type.

Unilateral ureteral obstruction (UUO) is a model of progressive renal fibrosis in rodents and is known to mimic accelerated human chronic obstructive nephropathy with primary injury in renal tubules caused by obstructed urine flow.^68^ Three studies have shown increased expression of KLF5 mRNA and/or protein in the unilateral obstructed kidney compared to contralateral unobstructed kidney of mice with UUO.^12^, ^13^, ^69^ When the C57BL/6 mice subjected to UUO, the expression of KLF5 was induced in renal tubule cells of the unilateral obstructed kidney (both cortex and medulla) as compared to the control kidney, which were detected by Western blot and immunohistochemistry staining, along with increased proximal tubular cell proliferation and possible progression of kidney fibrosis.^13^ Furthermore, immunofluorescent staining revealed the staining of KLF5 was mainly in proximal tubules of fibrotic kidneys, but not in endothelial cells and fibroblasts.^13^ Zhou et al also revealed increased expression of KLF5 mRNAs in the unilateral obstructed kidney of UUO mice at 10 days after the operation.^69^

Subtotal 5/6 nephrectomy (5/6 Nx) is another commonly used experimental model of chronic renal failure. Li *et al* subjected male C57BL/6 mice to 5/6 nephrectomy, which demonstrated significantly increased renal fibrosis and renal dysfunction (increased ACR and BUN) at 12 weeks after operation, along with significant increase in renal expression of KLF5, predominantly in the renal tubular cells as detected by immunofluorescent staining and Western blotting.^11^ In the fibrotic kidneys induced by 5/6 nephrectomy, Chen *et al* (2015) reported that KLF5 expression was increased in both the renal cortex and medulla regions, but not in endothelial cells and fibroblasts, these results were consistent with what was demonstrated in UUO‐induced renal fibrosis and dysfunction.^13^

Increased KLF5 expression was also observed in diabetic kidney and diabetic kidney disease is associated with morphological changes including expansion of mesangial matrix and tubular interstitial space, as well as podocyte damage and glomerular basement membrane thickening.^10^, ^70^ Western blot analysis demonstrated increased expression of KLF5 protein in the renal cortex of db/db mice (9‐weeks of age), compared to control mice.^10^ As summarized in Table 2, increased renal expression of KLF5 was seen in other animal models of various aetiological kidney diseases, for instance, in the renal medulla of spontaneously hypertensive rats, in which the mRNA expression of renal KLF5 was increased along with the progression of renal epithelial‐mesenchymal transition^71^; in the kidneys of mice irradiated at the renal region with single dose of 16 Gy, in which, renal tissues were collected at the 10 and 20‐week time‐point after irradiation and showed a significant increase in KLF5 mRNA.^66^

In addition to KLF5’s role in modulating chronic kidney injury, KLF5 was also shown to regulate acute renal pathogenesis such as the nephritis (Table 2). Huang *et al* fed rats melamine and cyanuric acid (M/CA) for 3 days to generate the crystal‐induced nephritis. These rats that fed on M/CA, exhibited acute kidney injury including, increased blood BUN, crystal deposition, renal tubular cell damage and infiltration of macrophages along with an increased expression of KLF5 in renal tubule cells, especially when crystals were deposited within the lumen.^72^

Based on the above discussion, it is clear that renal expression of KLF5 is increased in several renal disease models, including chronic and acute diseases and its expression is associated with the renal pathogenic progression such as fibrosis and dysfunction. However, most of the studies mentioned above and also summarized in Table 2, only exhibited their association. Further studies are needed to examine whether increased KLF5 expression is causative and essential in these acute and chronic renal pathogenesis associated fibrosis and dysfunction. Fujiu *et al* ^12^ examined the role of KLF5 in regulating tubulointerstitial injury of the kidney induced by UUO in C57BL6/6J (wild‐type, WT) mice or in Klf5 haploinsufficient (Klf5^+/−^) mice on the same genetic background as WT mice, as Klf5 knockout (Klf5^−/−^) mice were found to be embryological lethal.^73^ This study showed that in the control WT mice, KLF5 was expressed in the nucleus of collecting duct cells. However, after initiation of UUO, KLF5 expression was markedly up‐regulated in the collecting duct cells and KLF5 was also be observed in a few of interstitial cells at 10 days after UUO. There were no functional or structural changes in the kidneys of Klf5^+/−^ mice. However, when UUO was performed on Klf5^+/−^ mice, they had less tubular structural injury, decreased macrophage accumulation and pro‐inflammatory cytokines, but more severe interstitial fibrosis than WT UUO mice. Therefore, these authors concluded that their results clearly indicated that the collecting duct is an essential modulator of renal inflammation and dissecting these underlying molecular mechanisms further may lead to the identification of targets for generation of novel therapies.

In general, studies from above animal models of renal diseases have strongly suggested the involvement of KLF5 in these diverse aetiological renal diseases, in support of which, human renal tissues from different pathogenic conditions also demonstrated increased renal expression of KLF5 in response to pathogenic stimuli.^74^ For instance, Cippa *et al* analysed cellular responses to ischaemia and reperfusion in 163 biopsies from a total of 42 human transplanted kidneys at 4 time‐points (before or after transplantation) by RNA‐seq transcriptional profiling.^74^ KLF5 was one of the genes markedly up‐regulated after transplantation.^74^ Moreover, in a separate study, a clinical observation showed that KLF5 expression was decreased in tumour tissues of ccRCC patients and that a better prognosis outcome was associated with increased expression of KLF5.^37^ Additionally, KLF5 mRNA levels were decreased in the peripheral blood mononuclear cells from paediatric nephrotic syndrome patients compared with control individuals.^75^

In summary, although the KLF5 pathogenic mechanisms of various renal diseases have not been well characterized, its involvements in several renal diseases have clearly emerged. Therefore, a systemic investigation of KLF5’s roles in these renal diseases and potential mechanisms underlying its actions is urgently needed and this may provide important novel targets for the prevention and treatment of some renal diseases.

## 4 THE CURRENT AND POSSIBLE FUNCTIONS, REGULATORY MECHANISMS OF KLF5 ACTING ON VARIOUS KIDNEY DISEASES

The studies in the kidney, demonstrate that KLF5 modulates kidney diseases by regulating a variety of cellular responses including apoptotic cell death, inflammation, cell proliferation and fibrosis, making KLF5 a plausible therapeutic drug target to treat kidney diseases.

**FIGURE 1:**
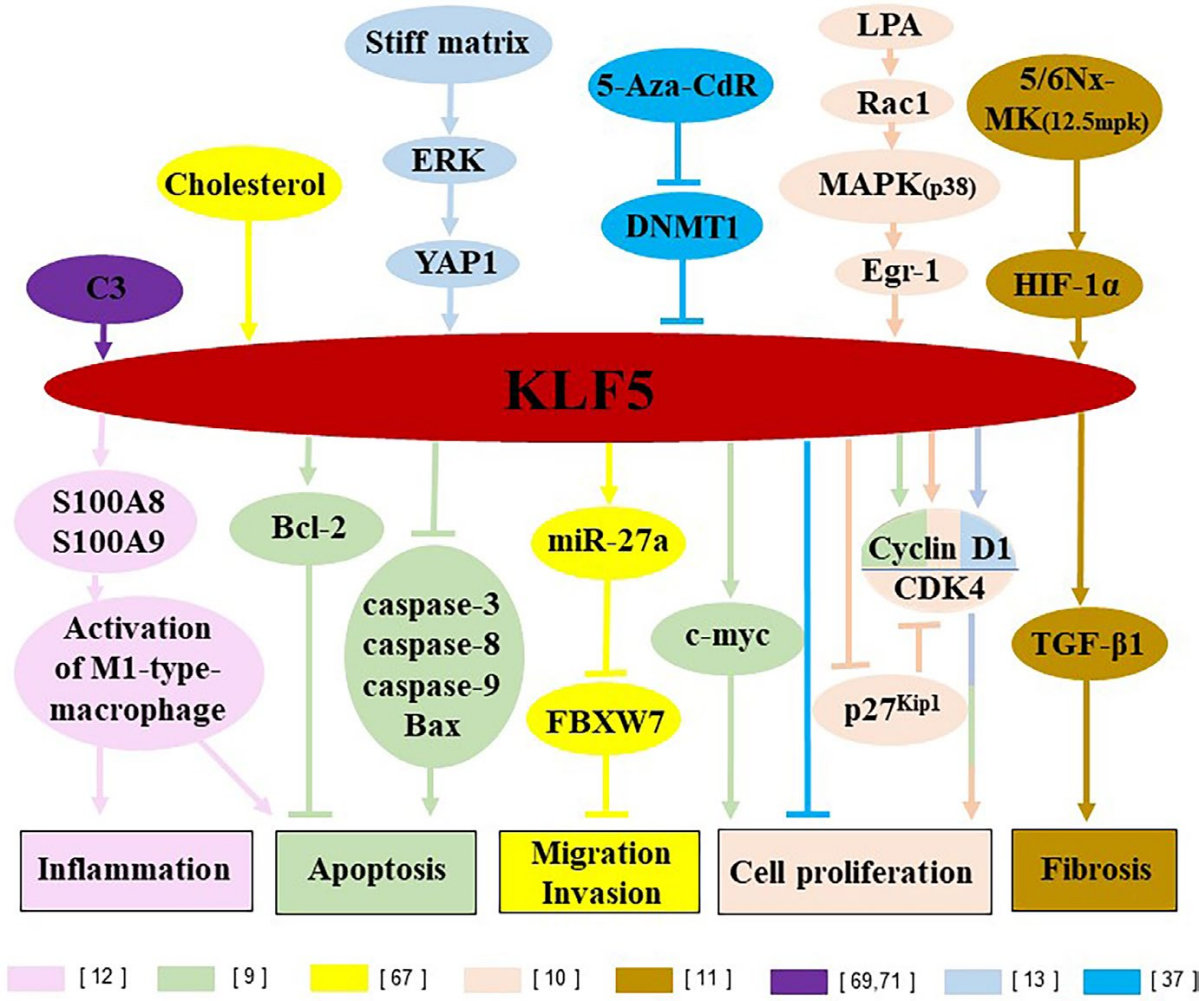
Current mechanisms underlying KLF5 effects on kidney diseases. KLF5 can regulate inflammation, apoptosis, fibrosis and cell proliferation in kidney via activation/inhibition of a variety of pathways, as detailed in the current review article. Various signalling pathways modulated by KLF5 are colour‐coded and references attached to each pathway are included in the figure

In the following sections, we have summarized the role of KLF5 in modulating apoptosis, cell proliferation, inflammation, oxidative stress, obesity/diabetes, fibrosis, stemness and differentiation in these acute and chronic renal diseases, as potential mechanisms contributing to these kidney diseases. The summarized potential mechanistic pathways of KLF5 in these kidney diseases are illustrated in Figure 1, including the roles of KLF5 in various cell types in the kidney.

### 4.1 KLF5 and apoptosis

Apoptosis is usually the cellular response to injury or its microenvironment alterations and is associated with the activation of cell death mediators and an inactivation of pro‐survival factors. Renal tubular cells and glomerular podocyte apoptosis contribute to parenchymal cell loss during acute or chronic kidney injury.^76^ Podocyte loss can lead to proteinuria and contribute to renal failure.^77^ Li *et al* have examined the role of KLF5 on podocyte apoptosis in vitro.^9^ They treated podocytes with puromycin aminonucleoside (PAN, 60 µg/mL), known to cause podocyte injury, for 6 hours, the results of flow cytometry demonstrated that the number of apoptotic podocytes was markedly increased in PAN group compared to control group, but overexpression of KLF5 by transfection with pCDNA3.1‐KLF5 plasmid in podocytes decreased the number of PAN‐induced apoptotic podocytes. Overexpression of KLF5 in PAN‐treated podocytes, decreased the expression of apoptosis‐inducing proteins Bax, caspase‐3, caspase‐8, caspase‐9 and increased the expression of anti‐apoptotic protein Bcl‐2. Therefore, overexpression of KLF5 in podocytes inhibited PAN‐induced podocyte apoptosis possibly by modulating expression of apoptosis regulating proteins. However, this study was performed with a cell culture model and so far, there are no studies performed in animal models of kidney disease that have explored the expression of KLF5 in podocytes (Table 2). In contrast to this in vitro study, an in vivo study showed an opposite, pro‐apoptotic role for KLF5 on renal cell apoptosis.^12^ These authors used a mouse UUO‐model of kidney fibrosis, where immunostaining experimentation showed that UUO led to up‐regulation of KLF5 compared to control group at 4, 10 days after UUO in kidney of WT mice. Meanwhile, TUNEL staining demonstrated significantly decreased renal cell apoptosis in the renal cortex and medulla of UUO‐treated haploinsufficient KLF5^+/−^ mice as compared to UUO‐treated WT mice. CD11b^+^F4/80^lo^ cells are the hallmark of pro‐inflammatory type 1 (M1) macrophage phenotype. Flow cytometry analysis revealed decreased CD11b^+^F4/80^lo^ cells in KLF5^+/−^‐UUO mice compared to WT‐UUO mice. Additionally, these authors demonstrated that overexpression of KLF5 in murine inner medullary collecting duct mIMCD‐3 cells promoted accumulation of M1‐activated macrophages. Culturing mIMCD‐3 cells with medium conditioned by CD11b^+^F4/80^lo^ cells had enhanced apoptotic cells compared to control group. Collectively these studies revealed that KLF5 promoted renal cell apoptosis by inducing accumulation and activation of M1‐macrophages while a protective role for KLF5 in PAN‐induced podocyte apoptosis was also documented by modulating expression of apoptosis regulating proteins. Thus, KLF5 could have divergent effects on apoptosis of different cell types in the kidney.

### 4.2 KLF5 and proliferation

Dysregulated proliferation of renal mesangial cells and tubular cells leads to glomerulosclerosis and tubulointerstitial fibrosis in kidney.^78^ The role of KLF5 in proliferation of renal mesangial cells and tubular cells has been identified.^10^, ^13^ As we know, mesangial expansion is one of the important features in diabetic kidney disease.^70^ Therefore, Kim *et al* tested the expression of KLF5 in kidneys of diabetic db/db mice and demonstrated that KLF5 protein expression was induced in the renal cortex of db/db mice (9 weeks of age) compared to control group as detected by Western blot.^10^ To further discuss the mechanism of KLF5 in the proliferation of renal mesangial cells in diabetic kidney disease, they performed in vitro cell experimentation. They treated mesangial cells (SV40 MES13) with lysophosphatidic acid (LPA), known to promote the proliferation of renal cells. These studies demonstrated an increase in the number of viable SV40 MES13 cells after 24 and 48 hours of LPA treatment. Moreover, both KLF5 mRNA and protein expression increased in SV40 MES13 cells after 6 hours of LPA treatment, along with increased expression of cell proliferation indicators (cyclin D1 and CDK4) and decreased expression of CDK inhibitor (p27^Kip1^) at the 12 hours LPA time‐point. However, it is important to note that these authors demonstrated a decrease in KLF5 expression in SV40 MES13 cells after 12 hours of LPA treatment and at the same time‐point they demonstrated an increase in cyclin D1 and CDK4 expression and a decrease in p27^Kip1^ expression. Furthermore, a pro‐proliferative role for KLF5 was confirmed by silencing KLF5 expression in SV40 MES13 cells. They demonstrated that knocking down KLF5 with siRNA in SV40 MES13 cells up‐regulated the expression of p27^Kip1^ and decreased the number of viable cells compared to control group. As a result, this study concluded that KLF5 promoted cell proliferation in LPA‐treated renal mesangial cells.^10^ In addition, Chen *et al* tested the role of KLF5 in regulating proliferation of renal tubular cells in vivo and in vitro.^13^ In UUO mouse kidneys, the results of Western blot and immunohistochemistry revealed increased expression of KLF5 and cyclin D1 as compared to control group. KLF5 and cyclin D1 expression were also increased in the mouse proximal tubular epithelial cells cultured on stiff matrix which was associated with promoting cell proliferation. Knocking down KLF5 by shRNA repressed the expression of cyclin D1 and blocked cell growth. These results indicated that KLF5 promoted proximal tubular cell proliferation by up‐regulating cyclin D1.^13^ Collectively, the above two studies demonstrated that KLF5 promoted renal mesangial and tubular cell proliferation. In contrast, a pro‐proliferative factor KLF5 was converted to an anti‐proliferative factor in the presence of TGF‐β in an epithelial cell line.^20^ In the absence of TGF‐β, KLF5 was shown to inhibit CDKN2B, a cell cycle inhibitor, while in the presence of TGF‐β, KLF5 induced CDKN2B expression and blocked cell proliferation.^20^, ^79^ TGF‐β is a critical mediator of diabetes‐induced kidney fibrosis.^80^, ^81^ Therefore, in the presence of TGF‐β, the effect of KLF5 on renal cell proliferation needs to be further studied in the context of diabetic kidney disease.

### 4.3 KLF5 and inflammation

Inflammatory response is widespread in acute and chronic kidney injury and it contributes to the progression and outcome of kidney injury.^82^ KLF5 promotes inflammatory response in kidney disease by promoting activation of pro‐inflammatory M1‐type macrophages. This was demonstrated in UUO‐induced kidney injury and melamine‐cyanurate crystal‐induced acute nephritis.^12^, ^72^ Huang *et al* demonstrated increased mRNA and protein expression of KLF5 in the rat kidney of crystal‐induced nephritis by performing RT‐PCR and immunohistochemistry staining. They also documented increased mRNA expression of pro‐inflammatory mediators (IL‐1, MCP‐1) in 3‐week‐old rats, following which they demonstrated the accumulation of macrophages as demonstrated by immunostaining for the macrophage marker, ED‐1. Thus, increased KLF5 expression may regulate tubulointerstitial inflammation.^72^ Fujiu *et al* further provided a direct link between KLF5 and renal inflammation. As we mentioned above, UUO led to up‐regulation of KLF5 expression and accumulation of macrophages. KLF5 promoted the activation of pro‐inflammatory M1‐macrophages in UUO‐induced kidney injury. Meanwhile, UUO‐treated haploinsufficient KLF5^+/−^ mice reduced expression of tnfα and il1b genes, known to encode for pro‐inflammatory factors TNF‐α and IL‐1β as compared to UUO‐treated WT mice. As a result, KLF5 induced renal inflammation by promoting activation of pro‐inflammatory M1‐macrophages in UUO‐induced kidney injury.^12^ Moreover, chemotactic peptides S100A8/S100A9 were induced by KLF5 and exogenous administration of recombinant S100A8/S100A9 peptides into mouse kidneys promoted an accumulation of CD11b^+^F4/80^lo^ cells as detected by flow cytometry. Collectively, KLF5‐S100A8/S100A9‐M1‐macrophages pathway promoted renal inflammation in UUO‐induced kidney injury.^12^ Thus, the above studies demonstrate a role for KLF5 in modulating inflammation in the kidney. However, the precise mechanisms underlying these effects are not known. In addition to the kidney, the role of KLF5 on regulating inflammation is widely studied in other organs and the possible mechanisms mediating these effects have been discussed below. These mechanisms may provide some future directions in delineating mechanisms by which KLF5 modulates renal inflammation.

KLF5 can play dual roles in modulating inflammation. The pro‐inflammatory effect of KLF5 was verified in vivo and in vitro.^12^, ^83^, ^84^, ^85^, ^86^, ^87^, ^88^ KLF5 induced expression of pro‐inflammatory cytokines namely, TNF‐α, IL‐1β and IL‐6, by activating NF‐κB pathway. KLF5 was also shown to directly regulate the transcriptional activation of these pro‐inflammatory genes.^83^, ^86^, ^87^, ^88^ In addition, pro‐inflammatory TNF‐α was also shown to induce KLF5 expression in human VSMCs.^51^ Chanchevalap *et al* demonstrated that KLF5 promoted inflammation *via* activation of the LPS‐MAPK‐KLF5‐NFκB pathway in LPS treated IEC6 cells. IEC6 cells treated with LPS demonstrated an increase in expression of KLF5, NF‐κB subunits (P50, P65), TNF‐α, IL‐6 and ICAM‐1. Pretreatment of IEC6 cells with U0126, a MEK1/2 inhibitor, prior to stimulation with LPS, inhibited the aforementioned mRNA induction. Moreover, silencing KLF5 expression in IEC6 cells inhibited secretion of pro‐inflammatory mediators TNF‐α and IL‐6.^84^ KLF5 induced pro‐inflammatory cytokine expression and modulated M1/M2 macrophage recruitment.^12^ Inhibition of KLF5 expression with siRNA suppressed pro‐inflammatory TNFα‐induced monocyte chemoattractant protein‐1 (MCP1) expression in human umbilical vein endothelial cells (HUVECs).^85^

An anti‐inflammatory role for KLF5 is also reported. KLF5 could repress the expression of various pro‐inflammatory mediators, such as TNF‐α, IL‐1β. Li *et al* demonstrated that KLF5 exerted its anti‐inflammatory role through modulation of PPAR‐γ/PGC‐1α/TNF‐α signalling pathway in the H9C2 cells, a model of myocardial oxygen‐glucose deprivation/reperfusion (OGD/Rep) injury. In this model, they demonstrated decreased KLF5 expression concurrent with increased expression of TNF‐α, IL‐1β, IL‐6 and IL‐8. In contrast, overexpression of KLF5 in H9C2 cells resulted in down‐regulating expression of these pro‐inflammatory cytokines by increasing expression of PPAR‐γ/PGC‐1α. Pretreatment with GW9662, a PPAR‐γ selective antagonist, prevented effects of KLF5 overexpression on PPAR‐γ, PGC‐1α and TNF‐α expression.^89^ These results suggest that the protective role of KLF5 in alleviating OGD/Rep injury occurs through regulation of PPAR‐γ/PGC‐1α/TNF‐α pathway. Further studies in the kidney are needed to delineate the mechanisms by which KLF5 regulates inflammation in a variety of kidney diseases and identifying these precise mechanisms may lead to generation of therapies to control inflammation‐mediated kidney injury/disease.

### 4.4 KLF5 and oxidative stress

Although oxidative stress promotes inflammation, apoptosis and fibrosis in kidney disease,^90^ no study to date has examined a direct link between oxidative stress and KLF5 expression in the kidneys. However, a role for KLF5 modulation by oxidative stress is documented in different cells and organ systems. These studies may provide a direction for future studies in the kidney.

KLF5 can regulate oxidative stress and vice versa. Zhang et al showed that KLF5 could promote oxidative stress by targeting glutathione‐S‐transferase Mu 1 (Gstm1) to regulate glutathione metabolism.^91^ While another study demonstrated that oxidative stress leads to activation of insulin‐KLF5 pathway. In this study it was demonstrated that insulin stimulation of HUVEC cells increased KLF5 mRNA expression which was subsequently inhibited in the presence of reactive oxygen species inhibitor (DPI), suggesting a role for oxidative stress in inducing KLF5 mRNA expression.^92^ Similarly, in VSMCs, preincubation of cells with DPI, inhibited Ang II‐induced KLF5 expression.^93^ Moreover, oxidative/nitrosative stress‐induced KLF5 expression and translocation into nuclei in lung fibroblasts. In this study, stimulation of human foetal lung fibroblasts (HFL‐1) with H_2_O_2_ or ONOO^−^ induced KLF5 expression in a dose‐dependent manner.^94^

### 4.5 KLF5 and obesity, diabetes

Obesity and diabetes are the major risk factors for the development of kidney diseases and KLF5 activation is modulated in obesity and in diabetes. KLF5 can regulate adipocyte differentiation as dominant negative KLF5 overexpression inhibited, while overexpression of wild‐type KLF5 induced adipocyte differentiation. Moreover, adipocyte differentiation was markedly attenuated in neonatal heterozygous KLF5 knockout mice. KLF5 exerted its effects *via* C/EBPβ/δ/PPARγ2 pathway.^52^ Additionally, KLF5 can also regulate energy metabolism. Sumoylated KLF5 repressed fatty acid oxidation genes, including, Ucp2, Ucp3 and Cpt1b, and the KLF5^+/−^ mice had more systemic energy expenditure. The KLF5^+/−^ mice were afforded protection from high‐fat‐induced obesity, hypercholesterolaemia and glucose intolerance.^34^ Moreover, genetic variation such as a TT Mutant Homozygote of KLF5 could increase basal metabolic rate and resting metabolic rate in Korean children.^95^ KLF5 was also found to regulate cardiac fatty acid metabolism via induction of peroxisome proliferator‐activated receptor α (PPAR‐α) expression. Cardiac PPAR‐α expression was decreased in cardiac myocyte‐specific KLF5 knockout mice along with a decrease in expression of its fatty acid metabolism‐related targets. This resulted in reduced cardiac fatty acid oxidation, ATP levels, along with increased triglyceride accumulation, and cardiac dysfunction.^96^

A clinical trial demonstrated that decreased fasting plasma glucose in type 2 diabetic patients treated with high‐performance inulin supplementation, was attributed to miR375‐KLF5 signalling.^97^ Moreover, inhibition of KLF5 could suppress TNF‐α induced MCP‐1 expression in HUVECs.^85^ Additionally, suppression of MCP‐1 demonstrated a minimal effect on glucose metabolism and insulin resistance on mice fed on a normal diet. While increased MCP‐1 expression in mice fed on high‐fat diet was associated with macrophage infiltration into adipose tissue, insulin resistance and hepatic steatosis.^98^, ^99^ Furthermore, Wang et al demonstrated that hyperinsulinaemia induced KLF5 expression in the endothelial cells from type 2 diabetic mice.^92^

### 4.6 KLF5 and fibrosis

Renal fibrosis is a direct consequence of kidney's inability to regenerate after kidney injury and ultimately leads to chronic kidney disease (CKD). KLF5 regulates fibrosis in a variety of organs including kidneys, heart, liver, skin and lungs. Some studies have implicated KLF5 as a pro‐fibrotic protein while others have identified it as an anti‐fibrotic protein. These differences could be attributed to different insults/stimulants/cell types/disease models and may involve different organs. KLF5 was shown to promote renal fibrosis. A hypoxia‐inducible factor (HIF)‐prolyl hydroxylase inhibitor, MK‐8617 (MK), dose dependently regulated renal fibrosis, in a 5/6 nephrectomy model of CKD. Protective effect of MK was demonstrated at lower concentrations while it exacerbated fibrosis at a higher dose by inducing KLF5 expression through the HIF‐1α‐KLF5‐TGF‐β signalling pathway. Silencing KLF5 by siRNA reduced the expression of TGFβ and fibronectin induced in high‐dose MK‐treated HK‐2 cells and alleviated renal fibrosis.^11^ Similarly, Ang II‐induced cardiac fibrosis was attenuated in haploinsufficient mice (Klf5^+/–^ mice) compared to control mice.^73^, ^88^ Additionally, dimethylnitrosamine induced significant increase in KLF5 mRNA expression and induced liver fibrosis, suggesting a role for KLF5 in the fibrotic response.^100^ In contrast, in Klf5^+/–^ UUO‐mice, the expression of fn1 and tgfβ1 gene which encode for FN and TGF‐β were increased concurrent with increased renal fibrosis as compared to UUO‐WT mice. However, Klf5^+/−^ UUO‐mice demonstrated decreased renal functional decline as compared to UUO‐WT mice.^12^ Additionally, in mice with the simultaneous knockdown of Klf5^+/−^ and Fli1^+/−^, a model of systemic sclerosis, demonstrated severe dermal fibrosis and pulmonary fibrosis. Moreover, bleomycin‐induced lung fibrosis was exacerbated in Klf5^+/−^ mice when compared to wild‐type. KLF5 also transcriptionally repressed CTGF expression.^38^ These studies collectively suggested a pro‐ as well as an anti‐fibrotic role for KLF5.

### 4.7 KLF5, stemness and differentiation

The last stage of various chronic kidney diseases is end‐stage renal disease (ESRD). Treatment options for CKD patients routinely involve a multidrug therapy which may slow down but cannot reverse the process leading to ESRD, thus necessitating renal replacement therapy, including dialysis and kidney transplantation in patients with ESRD.^101^ However, dialysis is not an ideal treatment option due to adverse effects of dialysis on patients’ quality of life and the exorbitant medical costs associated with it. The shortage of donor kidneys and incidence of organ rejection has also limited the use of kidney transplantation.^102^ Therefore, identifying new therapies that will slow down progression to ESRD or will prevent kidney disease in a way that prolongs patients’ lives and improves their quality of life is necessary. Stem cell therapy for renal regeneration has been proposed using pluripotent stem cells (PSCs), such as embryonic stem cells (ESCs), adipocyte‐derived mesenchymal stem cells and bone marrow‐derived stem cells. PSCs have unlimited self‐renewal capacity and the ability to differentiate into various types of cells including kidney lineage cells. Kidney organoids paved the way for kidney disease modelling, drug discovery and possibly for transplantation in the future.^102^ Induced pluripotent stem (iPS) cells, first reported by Takahashi et al, are a type of PSCs, reprogrammed from somatic cells by four‐transcription factors, including KLF4, OCT4, SOX2 and c‐Myc.^103^ Similarly, KLF4, OCT4, SOX2 and c‐Myc reprogrammed normal human kidney mesangial cells into iPS cells.^104^ KLF5 plays a role in stemness and can regulate cell differentiation as it was reported to replace KLF4 and augment reprogramming of somatic cells to iPS cells.^105^ KLF5 maintained self‐renewal and pluripotency of mouse ESCs.^106^, ^107^, ^108^ Mouse ESCs underwent differentiation by destabilizing its pluripotent or ground state by fibroblast growth factor‐induced pERK activation. Knocking out KLF5 in ESCs hyperactivated ERK pathway and induced ESC differentiation. Constitutive KLF5 expression blocked mouse ESCs differentiation and KLF5 overexpression was sufficient to convert epiblast stem cells into a naive pluripotent state.^109^, ^110^ Additionally, a recent phase 1a clinical trial was performed where patients with atherosclerotic renovascular disease were infused *via* their renal artery with autologous adipose‐derived mesenchymal stem cells. Blood flow, renal parameters, blood pressure and inflammatory markers were examined prior to infusion and 3‐months post‐infusion. Administration of autologous adipose‐derived mesenchymal stem cells increased blood flow and glomerular filtration rate while it decreased blood pressure and inflammation.^111^ Although a precise role for KLF5 in generation of adipose‐derived mesenchymal stem cells is unknown, KLF5 has been identified as an important regulator of adipocyte differentiation.^52^, ^112^

In AKI, renal tubule cells have the capacity to regenerate and repair itself by renal cell structural remodelling. One such mechanism of renal regeneration involves dedifferentiation and proliferation of surviving renal tubule cells after AKI.^113^, ^114^ Interestingly, KLF5 was reported to promote dedifferentiation and regeneration of crypt cells in intestinal epithelium after radiation injury as KLF5 can modulate proliferation and stemness.^115^ However, the direct role for KLF5 in regulating renal cell dedifferentiation remains to be determined.

## 5 CONCLUSIONS AND PERSPECTIVES

The current evidence has shown that KLF5 is an important modulator of kidney diseases. It can modulate many pathways including, apoptosis, proliferation, inflammation, oxidative stress, and fibrosis associated with obesity/diabetes‐induced nephropathy as well as other kidney diseases. KLF5 can inhibit/activate the aforementioned pathways, depending upon the different insults/stimulants/cell types/disease models used in the studies. However, thus far, the precise mechanisms underlying KLF5 actions on various kidney diseases are still limited. The role of KLF5 in modulating fibrotic kidney disease in type I and type II diabetes needs to be further evaluated. These studies may identify KLF5 as a new therapeutic target for the treatment of fibrotic kidney disease associated with diabetes.

## CONFLICT OF INTEREST

The authors declare no conflict of interest.

## AUTHOR CONTRIBUTIONS

**Jia Li:** Conceptualization (equal); Writing‐original draft (lead). **Liang Liu:** Visualization (equal); Writing‐original draft (supporting). **Wenqian Zhou:** Visualization (equal). **Lu Cai:** Writing‐review & editing (equal). **Zhonggao Xu:** Writing‐review & editing (equal). **Madhavi Jagdish Rane:** Conceptualization (equal); Writing‐review & editing (equal).
